# Hemispherical Microelectrode Array for Ex Vivo Retinal Neural Recording

**DOI:** 10.3390/mi11050538

**Published:** 2020-05-25

**Authors:** Yoonhee Ha, Hyun-Ji Yoo, Soowon Shin, Sang Beom Jun

**Affiliations:** 1 Department of Electronic and Electrical Engineering, Ewha Womans University, Seoul 03760, Korea; yoonhee0127@gmail.com (Y.H.); dibbe@ewhain.net (H.-J.Y.); 2 Department of Bioengineering, TODOC Co., Ltd., Seoul 08394, Korea; swshin@to-doc.com; 3 Department of Brain and Cognitive Sciences, Ewha Womans University, Seoul 03760, Korea

**Keywords:** microelectrode array (MEA), hemispherical, retina, neural recording

## Abstract

To investigate the neuronal visual encoding process in the retina, researchers have performed in vitro and ex vivo electrophysiological experiments using animal retinal tissues. The microelectrode array (MEA) has become a key component in retinal experiments because it enables simultaneous neural recording from a population of retinal neurons. However, in most retinal experiments, it is inevitable that the retinal tissue is flattened on the planar MEA, becoming deformed from the original hemispherical shape. During the tissue deforming process, the retina is subjected to mechanical stress, which can induce abnormal physiological conditions. To overcome this problem, in this study, we propose a hemispherical MEA with a curvature that allows retinal tissues to adhere closely to electrodes without tissue deformation. The electrode array is fabricated by stretching a thin, flexible polydimethylsiloxane (PDMS) electrode layer onto a hemispherical substrate. To form micro patterns of electrodes, laser processing is employed instead of conventional thin-film microfabrication processes. The feasibility for neural recording from retinal tissues using this array is shown by conducting ex vivo retinal experiments. We anticipate that the proposed techniques for hemispherical MEAs can be utilized not only for ex vivo retinal studies but also for various flexible electronics.

## 1. Introduction

Recently, the restoration of sight has been enabled by implantable visual prosthetic systems for patients blinded by retinal degeneration such as retinitis pigmentosa (RP) or age-related macular degeneration (AMD) [1,2,3,4,5]. Although the retinal prostheses has already been attempted for clinical purposes, it is still a great challenge to improve the spatial and temporal resolution of prosthetic systems to those of normal vision [6,7,8]. This is not just because visual encoding in the retina is complicated, but also because the electrical stimulation strategy has not yet been optimized for prosthetic vision. To study retinal signaling and to improve the spatial and temporal resolution of electrical stimulation on the retina, various ex vivo and in vitro experiments have been performed using microelectrode arrays (MEAs) capable of stimulating and recording from individual retinal neurons [6,9,10].

**Figure 1:**
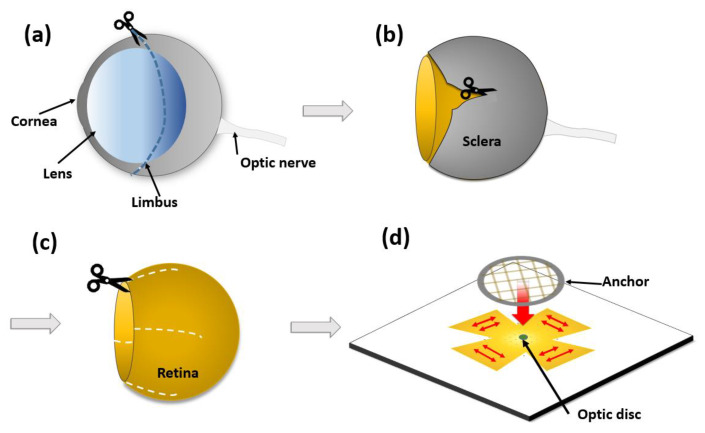
Overview of retinal preparation for ex vivo neural recording on a planar-type microelectrode array (MEA). (**a**) Cornea and sclera are divided along the limbus. (**b**) Sclera is cut to isolate the retina. (**c**) Retina is cut to facilitate flattening. (**d**) Retina is pressed down by anchor to adhere to the planar-type MEA.

The typical planar-type MEAs used for retinal studies have several tens of microelectrodes embedded in a transparent glass or polymer substrate [11]. To record neural activities from nearby retinal neurons through the microelectrodes, it is very crucial to locate the retinal tissues in the proximity of the electrodes. However, because of the mismatch between the flat surface of the MEAs and the curved shape of retinal tissues, skillful manipulation is required to closely attach the retinal tissue to the MEAs (Figure 1). In most studies of retinal tissues, to uniformly and closely attach the retina to the MEA, the tissue is stretched, flattened, and attached to a punched filter paper or anchored by mesh and pushed flat as shown in Figure 1d. During these procedures, the applied mechanical stress may induce abnormal physiological reactions. For instance, it was reported that mechanical pressure significantly increased the cellular apoptosis in animal ganglion cell lines as well as in human retinas, indicating acute or chronic glaucoma conditions [12,13,14]. Additionally, there have been several studies reporting that apoptosis of retinal ganglion cells is induced by increased intraocular pressure, which is another form of mechanical deformation [15,16]. As a direct evidence, there was a study reporting that the weight of anchors affect the stability of neural recording from retinal ganglion neurons [17]. It is also known that mice with a mutation for retinal degeneration have a much thinner retina structure than wild-type mice. For example, many researchers have used the rd1 mouse with a mutation in the Pde6b gene since it is the first strain of mouse identified with a retinal degeneration [18,19,20]. The retinas of rd1 adult mice are approximately two times thinner than those of wild-type mice, making them more vulnerable to mechanical stress and difficult to handle [21,22]. The effect of apoptosis via pressure is more sensitive in retinas from these mice than in normal retinas.

To overcome these problems, we propose a hemispherical MEA which is three-dimensionally conformal to the curved retina shape. The extracted retinal tissues can be attached to the proposed MEA without tissue deformation or mechanical stress to form stable connections between the microelectrodes and retinal ganglion cells (RGCs). We fabricated MEAs optimized to the retinal tissues of C57BL/6 mice so that the system can be utilized for studies using a mutant mouse model with retinal degeneration [9,23,24]. In addition, utilizing laser patterning and metal foil, we adopted a simple, low-cost fabrication techniques compared with the conventional thin-film microfabrication processes used for typical microelectrodes. The use of picosecond laser and Pt-foil enabled a low-cost, fast and design-flexible fabrications with a sufficient spatial resolution to fabricate several micro-sized electrodes. Using the fabricated electrode, neuronal activity was successfully recorded from the retinas of rd1 mice for up to five hours, indicating that the proposed hemispherical MEAs can reliably record retinal nerve signals. In the future, the number of electrodes can be increased to record retinal activity with a high spatial density.

## 2. Materials and Methods

### 2.1. Overview of Hemispherical MEAs

**Figure 2:**
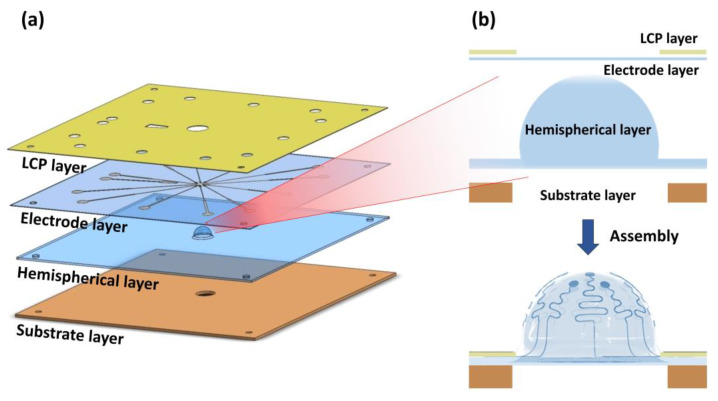
Structure of the hemispherical MEA. (**a**) Hemispherical MEA consisting of four layers (liquid crystal (LCP) layer, electrode layer, hemispherical layer, substrate layer). (**b**) Electrode layer is stretched into the shape of the hemispherical layer.

As shown in Figure 2, the hemispherical microelectrode array (MEA) consists of the following four layers in the order from top to bottom: liquid crystal polymer layer, electrode layer, hemispherical layer, and substrate layer. First, the liquid crystal polymer (LCP) layer is used as a stiff top layer to push down the thin, flexible electrode layer. The electrode layer is stretched to be conformal to the hemispherical layer which is the exposed bottom surface during the ex vivo experiments. The second layer is the electrode layer which has a sandwich structure consisting of PDMS (polydimethylsiloxane)-Pt-PDMS. This PDMS insulates the Pt electrodes and lines. Pt is a well-known electrode material because of its superior electrochemical properties and biocompatibility. Further, being malleable and ductile, Pt is suitable for flexible and stretchable electrodes. The electrode layer is made up of a thin PDMS film (~100 μm), allowing it to be stretched until it is conformal to the underlying hemispherical layer. The third layer from the top is made by molding PDMS to have the optimal curvature to fit the retina of a C57BL/6 adult mouse. The bottom layer is a substrate layer made of conventional printed circuit board (PCB) material to support the layers above. The device is designed to be compatible with a MEA1060-Inv Amplifier (Multi Channel Systems MCS GmbH, Reutlingen, Germany). The dimensions of the device are 49 mm × 49 mm with a thickness of 0.8 mm. Alignment holes were drilled at the edges of each layer to align and assemble the four layers. The details of the electrode design and fabrication procedures are described in the following sections.

### 2.2. Electrode Array Design

**Figure 3:**
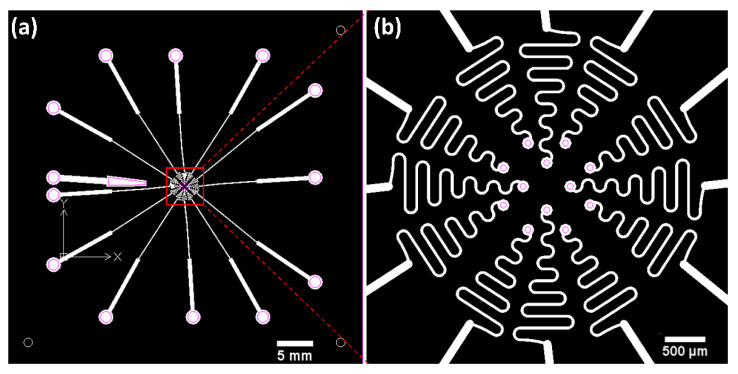
Two-dimensional electrode design before stretching into the hemispherical shape. (**a**) Overall view of electrode. A trapezoidal reference electrode is placed on the left. Thirteen connecting pads are located outside. (**b**) Zoomed-in image of the area including recording electrodes. Pink lines indicate the openings of the top polydimethylsiloxane (PDMS) insulation layer.

The electrodes are divided into four cardinal directions around the center location, and three electrodes are positioned in each direction. Therefore, a total of 12 channels of recording electrodes and a reference electrode are prepared as shown in Figure 3. The diameter of the recording electrodes is 150 μm, and a circular area with a diameter of 50 μm is exposed from the top PDMS insulation layer to provide an alignment margin between the patterned Pt conductor layer and the upper PDMS insulation layer. The width of the metal line is 30 μm, and the distances from the center to the electrodes are 300 and 500 μm (four electrodes at 300 μm and eight electrodes at 500 μm). The metal lines connected with the electrode sites are designed to have wavy patterns for stretchability and flexibility to allow for deformation into the retina-like hemispherical dome shape [25]. The radius of the wavy pattern is determined to be 150 μm by the equation from the literature, which can provide enough strain to be stretched into the hemispherical shape [26].

### 2.3. Fabrication of Hemispherical and Substrate Layers

The hemispherical layer is designed to be a dome-shaped structure with the same curvature as intact retinas of C57BL/6 adult mice. To determine the curvature of the adult mouse retina, different hemisphere sizes were prepared and extracted retinal tissues were directly placed on the hemispheres. The averaged radius of the mice retinas was determined to be approximately 1.52 mm which is consistent with the previous literature on wild type adult mice [27]. The radius of the hemispherical layer is set slightly larger (1.6 mm) to ensure a tight adhesion of the tissue.

**Figure 4:**
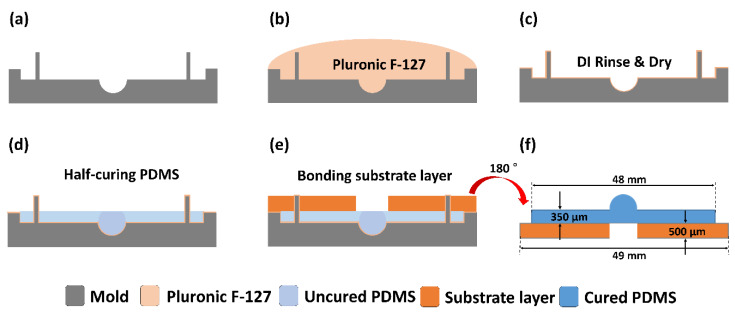
Fabrication of the hemispherical and substrate layers. (**a**) Mold with a concave hemispherical shape having the same size as mice retinas. (**b**) Surface treatment is performed to prevent adhesion between the stainless mold and PDMS. (**c**) Mold is rinsed with deionized water and dried with nitrogen. (**d**) PDMS solution is poured into the mold, degassed in the vacuum chamber, and half-cured in the oven. (**e**) The substrate layer is attached to the hemispherical layer and completely cured. (**f**) The hemispherical/substrate layers are released from the mold.

The PDMS hemispherical layer was molded from a stainless mold with a concave spherical shape in the middle as depicted in Figure 4a. Before molding the PDMS (Sylgard 184, Dow-corning, Midland, MI, USA), the mold surface was treated with a 5% nonionic surfactant (Pluronic F-127, Sigma-Aldrich, St. Louis, MO, USA) to facilitate PDMS release from the stainless mold after curing. The mold was soaked in the nonionic surfactant for one hour (Figure 4b), rinsed with deionized water, and dried with nitrogen (Figure 4c). After the surface treatment, 2 mL PDMS solution (Sylgard 184, Dow-corning, MI, USA) was poured to fill the mold. Then, the PDMS solution was degassed in a vacuum chamber and half-cured in a convection oven at 60 °C for 1 h (Figure 4d). After half-curing the PDMS, when the surface became sticky, a substrate layer made of plastic resin was attached to the PDMS hemispherical layer and cured in an oven at 80 °C for 30 min (Figure 4e). After the PDMS was completely cured, the hemispherical and substrate layers were carefully lifted together from the mold. The thicknesses of the hemispherical layer and the substrate layer were 350 μm and 500 μm, respectively. The substrate layer was designed to have a hole (3 mm in diameter) in the center for imaging with an inverted microscope (Figure 4f).

### 2.4. Fabrication of Electrode and Liquid Crystal Polymer (LCP) Layers

Of the total four layers, the top LCP layer and the thin PDMS electrode layer are fabricated together. The electrode layer is made by combining the bottom PDMS layers with metal electrodes and the top PDMS layer with opening holes as described in the following sections.

#### 2.4.1. Bottom Layer Fabrication

**Figure 5:**
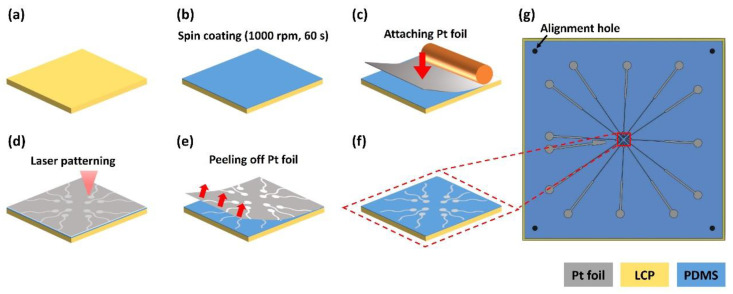
Fabrication of the bottom insulation layer including electrodes. (**a**) LCP is fixed on the wafer (wafer is not shown). (**b**) PDMS solution is spin-coated on the LCP and cured. (**c**) Pt foil is attached to the cured PDMS. (**d**) Pt electrodes are patterned by laser. (**e**) The background Pt foil is peeled off except for the electrodes and lines. (**f**) The bottom insulation layer with the LCP substrate is separated from the wafer. (**g**) Overall view of the bottom insulation layer (the center area including the electrode sites are enlarged in (**a**–**f**)).

The bottom layer was fabricated on a 4-inch silicon wafer. A 100 μm-thick LCP sheet (LCP, Vecstar CT-25, Kuraray, Japan) was trimmed to a 60 mm × 60 mm square and attached at its edge to the wafer with a commercial scotch tape (crystal clear tape, Scotch^®^, Saint Paul, MN, USA) (Figure 5a). On the LCP, a degassed PDMS solution was spin-coated at 1000 rpm for 60 s to form a 65-μm-thick PDMS film and cured at 60 °C for 1 h in the oven (Figure 5b). A 12.5 μm-thick Pt foil (Platinum Foil 99.95%, Goodfellow, Huntingdon, England) (50 mm × 50 mm) was attached to the cured PDMS using a hand roller (Φ32 × 50 mm rubber roller, HWA HONG Inc., Hwaseong, South Korea) to prevent bubbles (Figure 5c) [28,29,30]. The outlines of the electrode patterns were cut on the Pt foil using a pico-second laser (LPKF ProtoLaser R4, LPKF Laser & Electronics AG, Garbsen, Germany). The alignment holes were also cut through to the underlying LCP layer with the laser at increased power (Figure 5d). After laser patterning, the background Pt foil was carefully peeled off with forceps resulting in Pt electrodes on the PDMS layer (Figure 5e). After removing the background Pt foil, the bottom LCP layer was separated from the wafer (Figure 5f,g).

#### 2.4.2. Top Layer Fabrication

**Figure 6:**
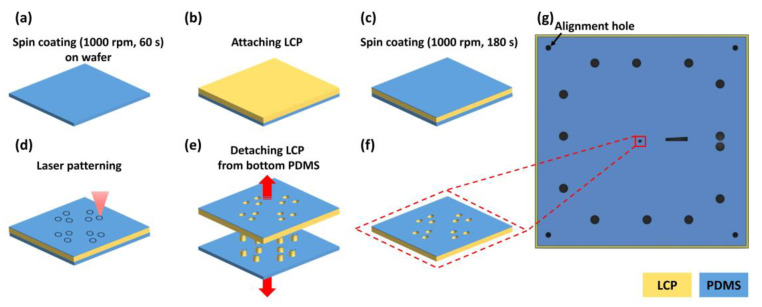
Fabrication of top insulation and LCP layers. (**a**) PDMS solution is spin-coated onto the wafer (wafer is not shown). (**b**) LCP is attached to the PDMS. (**c**) Second PDMS solution is spin-coated. (**d**) The outlines of electrode sites and alignment holes are cut by laser. (**e**) LCP is detached from bottom PDMS. (**f**) The top insulation of the electrode layer and LCP layer is completed. (**g**) Overall view of the top insulation layer. (The center area including the electrode sites are enlarged in (**a**–**f**)).

Like the fabrication of the bottom insulation layer, the top insulation layer was also fabricated on a 4-inch silicon wafer. First, a degassed PDMS solution was spin-coated at 1000 rpm for 60 s onto the wafer and cured at 80 °C for 30 min (Figure 6a). Then, 100-μm-thick LCP (60 mm × 60 mm) was placed on the PDMS surface and pushed down from one side to the other with a hand roller (HWA HONG Inc.) to flatten the layer without air bubbles (Figure 6b). After the LCP was attached, the second degassed PDMS solution was spin-coated onto the LCP (1000 rpm, 3 min) to form a 30-μm-thick PDMS layer and cured (80 °C, 30 min) (Figure 6c). The outlines of the electrode openings were cut using a laser through the upper PDMS and LCP layers without cutting the bottom PDMS layer (sacrificial layer) (Figure 6d). Then, detaching the LCP layers from the bottom PDMS layer resulted in clean electrode opening patterns because the cut circular patterns remained as attached on the bottom PDMS layer (Figure 6e). The patterned PDMS and LCP layers will be used as the upper insulation of the electrode layer and the top LCP layer.

#### 2.4.3. Bonding Between Top and Bottom Layers

**Figure 7:**
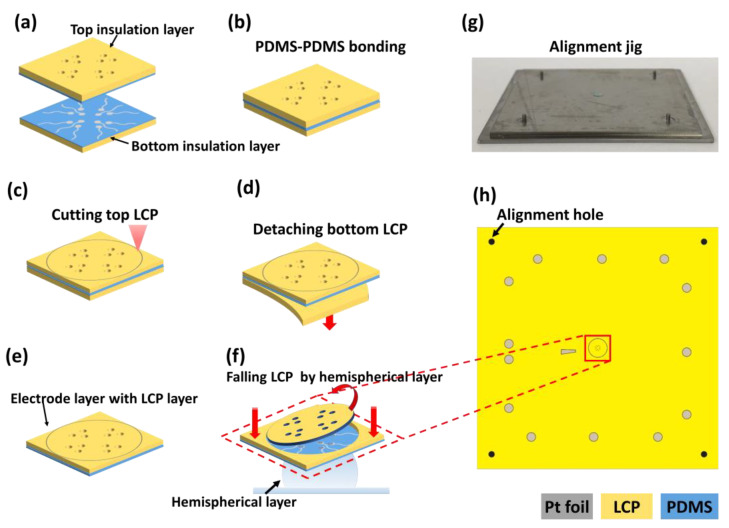
Bonding between top and bottom layers. (**a**) Top and bottom PDMS surfaces are activated and faced toward each other using a customized alignment jig. (**b**) PDMS-PDMS bonding is performed by pressure and heat. (**c**) A circle is cut into the top LCP using a laser. (**d**) Bottom LCP is removed from under the electrode layer. (**e**) Electrode layer with the LCP layer is completed. (**f**) Circles cut on the top LCP naturally fall off of the PDMS when assembled with the hemispherical layer. (**g**) Photograph of the custom alignment jig. (**h**) Overall view of electrode layer (the center area including the electrode sites are enlarged in (**a**–**f**)).

The electrode layer was completed by attaching the bottom and top layers via PDMS-PDMS bonding. After the PDMS surfaces of the bottom and top layers were activated by O2 plasma treatment (30 W, 15 s), the activated PDMS surfaces were aligned to face each other using a customized alignment jig (Figure 7a). The layers were gently pressed for ten seconds by hand. To increase the bonding strength, the layer was placed in an oven at 80 °C for 30 min [31,32]. After curing, a circle of 3.8 mm in diameter was cut into the top LCP using laser processing (Figure 7c). This circular area will later be used for deformation of the electrode layers into the hemispherical shape. Then, the bottom LCP is carefully removed from the electrode layer (Figure 7d), completing the electrode layer attached to the top LCP layer (Figure 7e). The circular-cut area on the top LCP will fall off from the PDMS electrode layer during the total assembly procedure, forming the hemispherical electrode layer (Figure 7f).

### 2.5. Mechanical Simulation of Hemispherical Stretching

Since most microfabrication technologies are based on two-dimensional thin film processes, it is almost impossible to directly fabricate three-dimensional microelectrode arrays. Therefore, we aimed to realize hemispherical MEAs by pressing the two-dimensional flexible electrode layer down onto the preformed hemispherical bottom layer as shown in Figure 2b. However, during this procedure, the thin PDMS electrode layer was easily torn, and sometimes the electrode metal lines were broken in spite of the wavy patterns. This was primarily because the electrode layer was not uniformly stretched because of the high friction between the electrode layer and the hemispherical layer. To quantitatively assess the effect of friction between two PDMS layers, mechanical simulations were performed to determine the strain distributions under dry and lubricated conditions between the two layers. To simplify the computational model, a two-dimensional simulation was performed using SolidWorks software (Dassault Systémes, Vélizy-Villacoublay, France). The coefficients of friction were 1 and 0.1 for dry and lubricated conditions, respectively, based on the literature [33]. 

### 2.6. Final Assembly for Hemispherical MEA

The hemispherical-substrate layer was fixed at the bottom of the alignment jig and corn oil (C8267, Sigma-Aldrich, St Louis, MO, USA) was applied to the surface of the hemisphere to reduce friction between the PDMS hemisphere and the PDMS electrode layer, as suggested by the mechanical simulation results. For adhesion between the electrode layer and hemispherical layer (except the circular electrode area), PDMS solution was applied to the hemispherical layer on the area outside of the hemisphere region. After aligning the LCP-electrode layer over the hemispherical-substrate layer, the LCP-electrode layer was pushed down to be stretched by the underlying hemispherical structure. When the center area of the electrode layer began to deform into the hemispherical shape, the circularly-cut LCP was detached from the PDMS surface (Figure 7f). After all layers were combined together, the assembled electrode was cured at 80 °C for 30 min. A plastic ring (diameter: 26 mm, height: 6 mm, thickness: 2 mm) was fabricated using a 3D printer (Form 2, Formlabs Inc., Somerville, MA, USA) and attached at the center of the MEA to form a chamber for ex vivo experiments.

### 2.7. Electrochemical Impedance Spectroscopy

To characterize the electrochemical properties of the fabricated microelectrodes, electrochemical impedance spectroscopy (EIS) was performed on each electrode in the frequency range of 1 Hz to 100 kHz in phosphate-buffered saline (PBS, Gibco, Waltham, MA, USA). The impedance was measured based on a three-electrode system using a potentiostat (ZIVE SP2, ZIVE lab, Seoul, South Korea). An Ag/AgCl electrode and a Pt wire were used as the reference and counter electrodes, respectively.

### 2.8. Ex Vivo Retinal Neural Recording

#### 2.8.1. Retina Preparation

An eight-week-old rd1 mouse (Pde6brd1, Woojung Bio, Suwon, South Korea) was anesthetized with isoflurane, and the eyeballs were extracted using forceps. The isolated eyeballs were washed in Ringer solution (124 mM NaCl, 5 mM KCl, 1.15 mM KH_2_PO_4_, 1.15 mM MgSO_4_, 10 mM glucose, 25 mM NaHCO_3_, 2.5 mM CaCl_2_) at 37 °C, which had been bubbled with 95% O_2_ and 5% CO_2_ gas for at least 30 min. The limbus was cut with a curved razor blade on a sterile gauze moistened with Ringer solution (Figure 1a), and the cornea was separated from the sclera using micro scissors. After removing the lens, the retina was separated from the sclera in Ringer solution (Figure 1b). The vitreous body in the retina was thoroughly removed with fine forceps because it can act as insulation if it remains. The isolated mouse retina was attached to the surface of the hemispherical MEA. The experimental procedures and the care of the animals were approved by the Institutional Animal Care and Use Committee (IACUC) of Ewha Womans University (No.20-024).

#### 2.8.2. Neural Recording

Spontaneous neural activity in the isolated retina was recorded on the hemispherical MEA. During the recording, Ringer solution was maintained at 33 °C using a temperature controller (TC02, Multi Channel System MCS GmbH) and continuously superfused with a peristaltic perfusion system (PPS2, Multi Channel System MCS GmbH) for long-term neural recording. The inlet and the outlet flow speeds were maintained at 0.4 and 0.6 mL/minute, respectively. The outlet tube was placed at two-thirds of the chamber height to prevent overflow of the solution. After stabilization for one hour, the neural activity was obtained and filtered with a second-order high-pass Butterworth filter (cutoff frequency: 200 Hz) to isolate the spike signals from the RGCs from slow wave components. The recorded neural activity was analyzed in terms of the spikes from individual RGCs using NeuroExplorer software (Plexon, Dallas, TX, United States). The spikes were detected using the amplitude threshold at -5 SD (standard deviation) of noise level.

## 3. Results

### 3.1. Mechanical Simulation of Hemispherical Stretching

**Figure 8:**
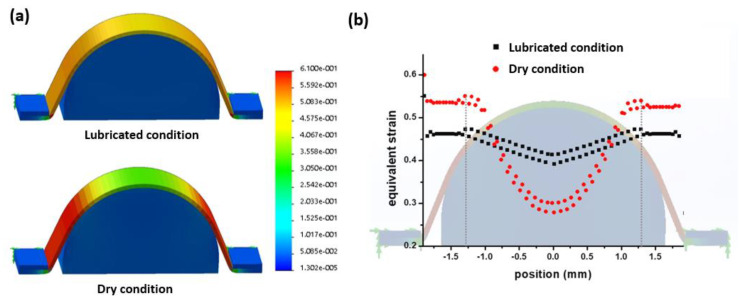
Two-dimensional simulation results for the strain of the stretched electrode layers under lubricated and dry conditions. (**a**) Strain color map of the electrode layer according to the surface conditions of the hemispherical layer. (**b**) Equivalent strain plots overlapped with the hemispherical electrode shape.

To verify the effect of the lubricant during hemispherical stretching of the electrode layer, the mechanical strains were evaluated under dry and lubricated conditions. The two-dimensional simulation clearly showed that the strain was more uniformly distributed under the lubricated condition. Under the dry condition, because of the high friction between the two PDMS layers, the strains at both edges were approximately two-fold higher than at the center region. The maximum strain under the dry condition was approximately 20% higher than the lubricated condition. As shown in Figure 8b, the position of maximum strain was 1.29 mm from the center position of the hemisphere. Tearing of the electrode layer frequently occurred at this position. Therefore, for the final assembly step, the lubricated condition was achieved by the application of corn oil between the electrode and the hemispherical layers, so the electrode layer could be uniformly stretched without tearing.

### 3.2. Electrode Fabrication

**Figure 9:**
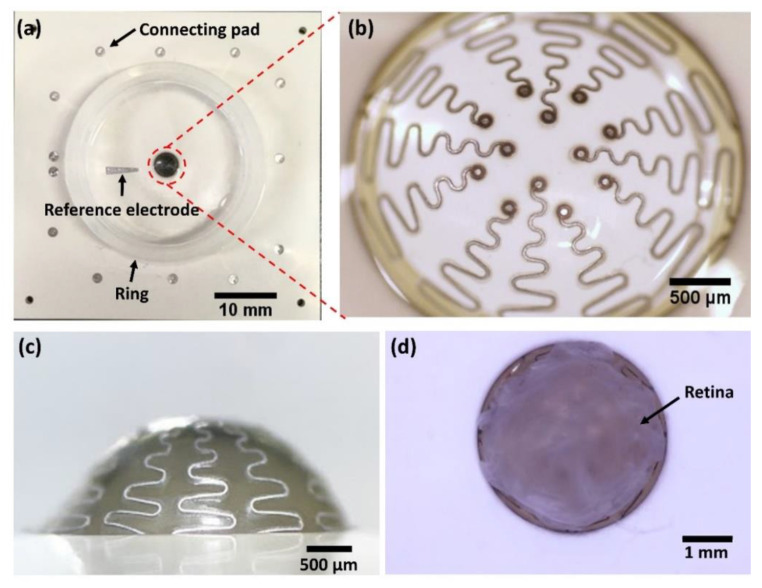
Fabricated hemispherical MEA. (**a**) Top view of the entire MEA. Twelve microelectrodes are located in the red circle. The trapezoidal reference electrode is on the left side of the chamber. The thirteen connecting pads are located outside of the chamber. (**b**) Zoomed-in top view of the hemispherical electrode area. (**c**) Side view of the hemispherical area. (**d**) Mounted whole retina on the hemispherical MEA.

A hemispherical MEA was successfully fabricated for ex vivo retinal neural recording as shown in Figure 9. The device consists of a reference electrode and 12 recording electrodes (Figure 9a,b). The electrodes were insulated by the top PDMS layer, and the electrode sites were exposed by a hole having a diameter of 50 μm (Figure 9b). Connected to the electrodes, 13 connecting pads are located outside of the recording chamber which is designed to fit a multichannel recording system (1060-Inv-BC amplifier, Multi Channel Systems MCS GmbH). A ring was attached to hold the Ringer solution. The recording electrodes are connected with connecting pads by wavy-patterned lines to prevent line breakage when stretched by the hemispherical layer. As shown in Figure 9b,c, the wavy lines were successfully stretched without breaking or tearing of the PDMS layer despite deformation from a flat circle into a hemispherical shape. The deformation led to approximately a two-fold increase of the area. When Pt/Ir alloy was used for the metal layer, the wavy patterns did not properly stretch, resulting in tearing of the top PDMS layers. Although the detailed results from different metals are not included in this section, the reasons for choosing Pt as the metal layer are described in the discussion section. It was also confirmed that a whole isolated retina from an rd1 mouse can be mounted on the hemispherical MEA as shown in Figure 9d. While the retinal tissue was immersed in the solution, the mounted tissue was stably attached without additional fixation because the size of the hemisphere is designed to be slightly larger than the actual retinal curvature.

**Figure 10:**
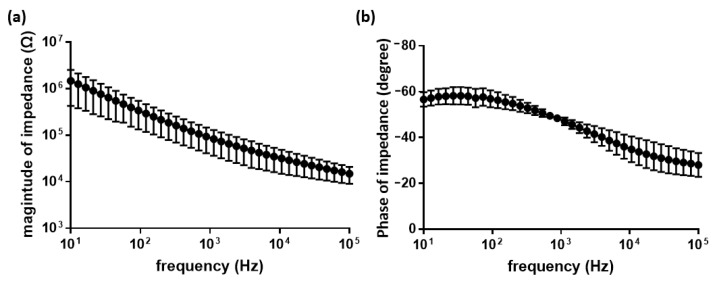
Electrochemical impedance spectroscopy of the fabricated hemispherical MEA. (**a**) Magnitude of impedance. (**b**) Phase of impedance.

Electrochemical spectroscopy showed that the fabricated electrodes have impedance characteristics appropriate for neural recording. Over the frequency range between 1 Hz and 100 kHz, the magnitude and phase of the impedance are plotted in Figure 10. Electrode impedance values of conventional planar-type MEAs (Multi Channel System MCS GmbH) are known to be approximately 100 kΩ for 30 µm TiN electrodes. The impedance value of the hemispherical MEA proposed in this paper was 87.5 ± 49.3 kΩ at 1 kHz, which is similar to that of conventional planar-type MEAs. The measured impedance value was appropriate for spike detection and sorting.

### 3.3. Ex Vivo Neural Recording from RGCs

**Figure 11:**
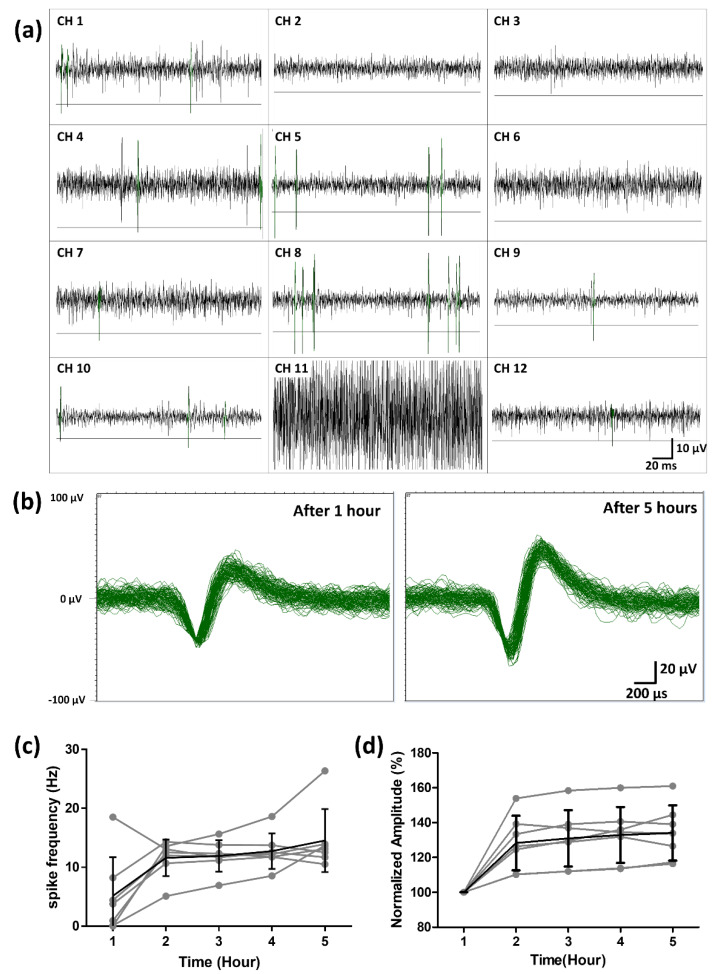
Spontaneous activity from rd1 mice retina recorded using the fabricated hemispherical MEA. (**a**) Neural signals from rd1 mice were measured in hemispherical MEAs. (The line in each channel is the value of -5 SD of the noise level for signal detection.) (**b**) The neural signals detected after 1 h (stabilization) and 5 h were overlaid in channel 4. (**c**) The spike frequency of the rd1 mice retina over five hours (the gray line is the spike frequency detected at each electrode. The black line is the average spike frequency of seven electrodes). (**d**) The normalized spike amplitude of the rd1 mice retina over five hours (the gray line is the normalized pike amplitude detected at each electrode. The black line is the average amplitude of seven electrodes).

After one hour of stabilization in the superfused Ringer solution, the spontaneous neural activity from the retinal ganglion cells (RGCs) was successfully recorded using the fabricated hemispherical MEA. From the waveforms without spike signals, the amplitudes of the background noise signals were measured to be approximately 15 μV peak-to-peak on average, except for one channel (Ch. 11) with a bad connection to the amplifier as shown in Figure 11a. For each channel, five times the standard deviation of the noise signals was used as the negative threshold level for spike detection. The threshold level for each channel is shown in Figure 11a. As an example, the detected neural spike waveforms after 1 h and 5 h from channel 4 were overlaid in the same time window in Figure 11b. In seven channels out of the twelve, the recorded neural spikes showed firing frequencies above 10 Hz during the five-hour recording session. The frequencies for each channel were plotted over time in Figure 11c. The frequency of each neural signal is shown in gray, and the averaged frequency in black. On average, the frequency of the spontaneous neural activity from the RGCs increased gradually during the first two hours and then stayed above 10 Hz. The means and the standard deviations of the firing frequencies were 5.14 ± 6.58 Hz after one hour and 14.5 ± 5.3 Hz after five hours. The spike frequency was similar to the value of the previous study which reported that the average frequency of rd1 RGCs is around 10 Hz when the retinas are extracted from P44-51 rd1 mice [34]. The percentage of normalized spike amplitude is plotted over five hours in Figure 11d. The amplitude of each neural signals is shown in gray and the averaged amplitude in black. On average, the amplitude of the spontaneous neural activity from RGCs increased gradually during the first two hours and then maintained above 125%.

## 4. Discussion

In this study, a hemispherical MEA suited for mice retinas is proposed. The MEAs are fabricated by sandwiching a thin, flexible electrode layer and a bottom layer with a hemispherical structure which is designed to fit the curvature of mice retinas. A rd1 mouse retina was attached to the fabricated MEA without deformation, and the spontaneous neural activity of the RGCs was successfully recorded. In typical setups for ex vivo retina experiments with planar-type MEAs, it is necessary to take advantage of additional techniques to closely attach the retina tissue to the microelectrode array. For example, the retina tissues must be flattened by pressing with a mesh-type weight, or they are forced onto perforated MEAs by underlying negative pressure. During these procedures, the retina tissues can be damaged and the mechanical deformation may induce abnormal physiological conditions such as apoptosis of the retinal neurons. However, the proposed hemispherical MEAs do not require excessive tissue deformation or mechanical pressure applied to the tissue. Therefore, it is expected that neural activity of RGCs can be monitored more stably under conditions close to those of the physiological environment. With the fabricated hemispherical MEAs, we showed that neural activity can be recorded for up to five hours, which is enough time for various ex vivo experiments. Further, the capabilities of the proposed hemispherical MEAs will be evaluated, especially for long term viability of the isolated retinal tissue, in comparison with conventional planar-type MEAs.

In this study, to verify the fabrication techniques, twelve electrodes were fabricated on a hemispherical MEA, which is a relatively small number of channels compared with conventional MEAs. As a next step, it is necessary to increase the number of channels on the electrode to measure the neural activity of the retina with higher spatial resolutions. However, increasing the number of channels requires narrower spacing of the electrode lines, which also reduces the radius of the wavy patterns due to the limited area. The reduced wavy patterns will inevitably increase the stress on the electrode lines. In addition, there is a limitation inherent in the laser processing. The electrode line spacing must be larger than 15 µm because of the laser spot size (15 µm). If the spacing is too narrow, it is difficult to remove the background Pt foil after laser patterning. It might be possible to dramatically increase the number of channels if thin film microfabrication processes are employed, such as metal deposition, photolithography, and etching. In this study, however, we aimed to utilize simple and low-cost fabrication techniques, such as the use of metal foil and laser patterning. Based on our methods, hemispherical MEAs can be fabricated with the increased number of channels, which is comparable to conventional MEAs. As a next step, we have designed a 52 channel electrode array with similar wavy line patterns. Because of the smaller line spacing and the reduced wavy patterns, it is expected to have difficulties in peeling-off of background Pt foils and stretching into the hemispherical shape. However, based on our simulation, the increased number of channels can be fabricated using same techniques described in this study.

The flexible electrode layer comprises thin PDMS layers and a laser-patterned Pt foil layer for insulation and the metal conductor, respectively. Platinum is a common conductor material for implantable electrodes for clinical applications, such as cochlear implants or deep brain stimulation systems, due to its superior biocompatibility and electrochemical properties. In this study, platinum is chosen because of its well-known advantageous properties for neural electrodes and its mechanical characteristics. Because of its high ductility compared to alternative metals, platinum is the most suitable for flexible electronics because wavy lines created from it can be easily stretched without tearing during the deformation of the electrode layer. We also attempted to fabricate the proposed hemispherical MEA using other metal foils, such as platinum-iridium alloy (Pt/Ir), silver, and aluminum. Pt/Ir is also a widely used biocompatible material in neuroprosthetic systems, but the electrode layer easily broke during deformation due to its low ductility. Aluminum is commercially available in various forms of foils, and the foil was compatible with the proposed fabrication processes. In addition, aluminum is the most common conductor material for integrated circuits. However, it was difficult to record neural activity with the Al electrode because of high electrochemical impedances. On the contrary, silver has good electrochemical properties, especially when it forms silver chloride. However, it is not chemically stable in physiological solutions and not stretchable once silver chloride is formed on the surface. Therefore, among these metal foil candidates, platinum is the most suitable for the flexible and stretchable conductor in this study.

In this paper, we focused on the fabrication of hemispherical MEAs and confirmed that it is possible to measure neural signals in the retinal tissues of rd1 mice using the fabricated three-dimensional MEAs. Even though the MEAs were designed to have twelve channels as a first step, the number of channels will be increased to perform simultaneous multi-channel ex vivo neural recording from the retinal tissue with a higher spatial resolution. As a further study, we will assess stress-induced retinal tissue responses by comparing long-term neural recordings from planer-type MEAs and hemispherical MEAs.

## 5. Conclusions

In the present study, we successfully developed hemispherical MEAs for ex vivo retina electrophysiological recording with minimal mechanical stress on the retinal tissue. The microelectrode array is designed to cover the adjacent area of the optic disc of the mouse retina. It is important to monitor the central area of the retinas because retinal ganglion neurons are densely distributed around the optic disc. In this study, the fabricated platinum electrodes, which ensure both mechanical and electrochemical stability as well as biocompatibility, were able to record spontaneous neural signals from RGCs for up to five hours. We expect that the proposed hemispherical MEAs will provide the advantages of simplified retinal preparation and stable long term monitoring. The proposed fabrication techniques can be also applied to flexible electronics which is an emerging technology for biomedical applications such as wearable sensors for health monitoring [35,36]. Electronic devices with high curvatures have been one of the biggest challenges in this field, and the proposed fabrication techniques can provide a solution to this problem.
