# Correction: Ha, Y. et al. Hemispherical Microelectrode Array for Ex Vivo Retinal Neural Recording. *Micromachines* 2020, *11*, 538

**DOI:** 10.3390/mi11080720

**Published:** 2020-07-24

**Authors:** Yoonhee Ha, Hyun-Ji Yoo, Soowon Shin, Sang Beom Jun

**Affiliations:** 1 Department of Electronic and Electrical Engineering, Ewha Womans University, Seoul 03760, Korea; yoonhee0127@gmail.com (Y.H.); dibbe@ewhain.net (H.-J.Y.); 2 Department of Bioengineering, TODOC Co., Ltd., Seoul 08394, Korea; swshin@to-doc.com; 3 Department of Brain and Cognitive Sciences, Ewha Womans University, Seoul 03760, Korea

The authors would like to make the following changes to the published paper [1]: the ethic code in the last line of Section 2.8.1 (page 8) should be “IACUC 19-025”:

“The experimental procedures and the care of the animals were approved by the Institutional Animal Care and Use Committee (IACUC) of EwhaWomans University (IACUC 19-025).”

The change does not affect the scientific results. The manuscript will be updated and the original version will remain online on the article webpage with a reference to this correction.
